# Development of a new clinical tool to evaluate the balance abilities of children with bilateral vestibular loss: The Geneva Balance Test

**DOI:** 10.3389/fneur.2023.1085926

**Published:** 2023-03-07

**Authors:** Emile Monin, Céline Bahim, Lou Baussand, Jean-François Cugnot, Maurizio Ranieri, Nils Guinand, Angélica Pérez Fornos, Hélène Cao Van

**Affiliations:** Division of Otorhinolaryngology (ORL) Head and Neck Surgery Institute, Clinical Neurosciences Department, University Hospital of Geneva (HUG), Geneva, Switzerland

**Keywords:** balance, children, vestibulopathy, cochlear implant, test, GBT

## Abstract

**Introduction:**

Vestibular deficits are considered rare in children, but the lack of systematic screening leads to underdiagnosis. It has been demonstrated that chronic vestibular dysfunction impacts the normal psychomotor development of children. Early identification is needed to allow for clinical management, ensuring better global development. For this purpose, our research group has developed the Geneva Balance Test (GBT), aiming to objectively quantify the balance capacity of children over a broad age range, to screen for bilateral vestibulopathy (BV), and to quantify the improvement of balance abilities in children.

**Methods:**

To determine the capacity of the GBT to quantify the balance capacity of children with BV, we conducted an observational prospective study with three populations: 11 children with BV, and two age-matched control groups composed of (1) 15 healthy subjects without the vestibular or auditory disorder (HS) and (2) 11 pediatric cochlear implant recipients (CIs) without vestibular disorders. Results of the three populations have been compared in three different age sub- groups (3–5, 6–9, and ≥10 years), and with results of a short, modified version of the Bruininks-Oseretsky test of Motor proficiency Ed. 2 (mBOT-2).

**Results:**

Statistical analyses demonstrated significant differences in the scores of the GBT between children aged 3–5, 6–9, and ≥10 years with BV and in both control populations (HS and CI). BV scores reflected poorer balance capacities at all ages. Children in the youngest CI sub-group (3–5 years) showed intermediate GBT scores but reached HS scores at 6–9 years, reflecting an improvement in their balance capacities. All the results of the GBT were significantly correlated with mBOT-2 results, although only a few BV completed the entire mBOT-2.

**Discussion:**

In this study, the GBT allowed quantifying balance deficits in children with BV. The BOT-2 test is not validated for children <4.5 years of age, and the GBT seems to be better tolerated in all populations than the mBOT-2. Furthermore, mBOT-2 results saturated, reaching maximum values by 6–9 years whereas the GBT did not, suggesting that the GBT could be a useful tool for monitoring the development of balance capacities with age and could be used in the follow-up of children with severe vestibular disorders.

## 1. Introduction

Dizziness, vertigo, and imbalance are frequent complaints in the adult population, and it is estimated that up to 30% of adults will present these symptoms at least once (1). However, these symptoms appear less common in the pediatric population, where current estimates suggest that 8% of children have presented dizziness (2). Nevertheless, systematic screening, even in the presence of symptoms, is rarely included in current clinical procedures. Consequently, the exact incidence of chronic vestibular disorders in the pediatric population and their impact on development remain unknown.

The few currently available studies show that in the pediatric population, transient dizziness is often benign, but chronic or progressive balance disorders of vestibular origin have a real impact on the psychomotor development of children (3). Moreover, mixed vestibulo-cochlear disorders are frequent, and more than a third of children with profound sensorineural deafness have vestibular disorders (4). This precise population endures a double sensory deficit, which means it is particularly at risk for developmental delays. To ensure better overall clinical management, potential vestibular disorders should be actively screened in children with hearing impairments, who are particularly at risk (5).

In the clinical field, semicircular canal and otolith function are evaluated using assessments of the vestibulo-ocular reflex and vestibular myogenic potentials. Although massively used, these tests only assess the function of single sub-units of the vestibular system independently. This does not completely represent the impact of vestibular impairments on the global balance function and/or the patient's ability to adapt to the vestibular deficit. Thus, no correlation has been established between the subjective symptoms of patients assessed with the Dizziness Handicap Inventory and their vestibular function tested by vHIT, caloric testing, o/cVEMP, or posturography (6). Interestingly, rotational testing seems to be most amenable in young children and best correlated with balance function. In addition, a moderate correlation has been found between the vestibulo-ocular reflex gain on the rotatory chair test and the results of the Bruininks-Oseretsky test of Motor proficiency Ed. 2 (BOT-2), which is the most widely used test for assessing balance in the pediatric population (4).

Although the above-mentioned diagnostic tests are easily performed in adults, they are restrictive and not always feasible in children. Consequently, children with balance disorders are often assessed using a global clinical evaluation, not always including objective measures of balance or vestibular function. In this context, the BOT-2 was demonstrated to be a sensitive and specific tool to screen for children with bilateral vestibulopathy (BV) (7). However, this test has only been validated for children aged between 4.5 and 12 years. A literature review found only a few other clinical tests evaluating the balance capacities of children. The Ghent Developmental Balance Test seems to be a useful tool for this purpose but is only validated until 5 years of age (8). A clinical tool, evaluating balance capacities over a broad age range, is lacking and could be useful in the identification of children with severe vestibular dysfunction and their follow-up.

In this context, our group has developed a new clinical test: the Geneva Balance Test (GBT) that integrated a playful dimension that can be easily accepted by young children, as soon as they can walk. Our main goal in designing the GBT was to create a test that could objectively measure balance capacities. This way, this test could be used as a screening test for severe vestibular dysfunction in children, such as BV, and could be used during follow-up to assess the evolution of balance abilities with age.

The hypotheses of this study are the following:

1. Children with BV should obtain significantly poorer scores at the GBT when compared to children in control groups.
2. The GBT and the mBOT-2 results should be in agreement in all tested subjects.
3. GBT scores should improve with age, providing useful information about the development of balance.

## 2. Method

### 2.1. Design and participants

The main objective of this study is to assess the ability of the GBT to quantify the balance capacity of children with BV compared and an age-matched population without vestibular dysfunction. To achieve this, an observational study was designed including a case and two control populations:

- Children with BV diagnosed following the Bárány consensus criteria (9) constituted the case population. They presented mixed vestibulo-cochlear disorders and were therefore cochlear implant or hearing aid users.
- A group of healthy subjects (HS) with no vestibular dysfunction and no hearing impairment constituted the first control group.
- A group of children with cochlear implant(s) (CI) without vestibular dysfunction constituted the second control group to exclude any involvement of hearing impairments and/or the cochlear implant in balance abilities.

A total of 37 children were included in the study: 15 HS, 11 BV, and 11 CI without vestibular disorders (see detailed demographic characteristics in Table 1). The children included were aged between 3 and 16 years and, due to the normal psychomotor development of children (11), we separated the three populations into three different age sub-groups. The youngest sub-group (for which the BOT-2 is not validated) was composed of 5 BV, 4 CI, and 5 HS who were 3–5 years of age. The second age sub-group included 3 BV, 2 CI, and 4 HS who were 6–9 years of age. The oldest sub-group included 3 BV, 5 CI, and 6 HS who were ≥10 years of age.

**Table 1:** Demographic characteristics of the participants included in the study.

**Study group**	**Age [years] at data collection**	**Biological gender**	**Hearing status *R***	**Hearing status *L***	**Etiologies**
BV 3–5 ans	5	Female	CI	CI	Waardenburg II
BV 3–5 ans	5	Male	CI	CI	Idiopathic
BV 3–5 ans	3	Male	CI	CI	CHARGE
BV 3–5 ans	4	Female	CI	CI	CMV
BV 3–5 ans	4	Male	Ext. hearing aid	Ext. hearing aid	CMV
BV 6–9 ans	7	Female	CI	CI	Idiopathic
BV 6–9 ans	6	Female	CI	CI	Usher
BV 6–9 ans	9	Female	Ext. hearing aid	Ext. hearing aid	CHARGE
BV ≥ 10 ans	10	Male	Ext. hearing aid	Ext. hearing aid	CHARGE
BV ≥ 10 ans	10	Male	CI	CI	Idiopathic
BV ≥ 10 ans	15	Male	CI	CI	Idiopathic
IC 3–5 ans	4	Female	CI	CI	Prematurity
IC 3–5 ans	3	Female	CI	CI	Usher
IC 3–5 ans	5	Male	CI	CI	CMV
IC 3–5 ans	4	Male	CI	CI	Idiopathic
IC 6–9 ans	8	Male	CI	CI	Idiopathic
IC 6–9 ans	8	Female	CI	CI	Idiopathic
IC ≥ 10 ans	10	Female	CI	CI	Prematurity
IC ≥ 10 ans	10	Male	CI	CI	Idiopathic
IC ≥ 10 ans	14	Male	CI	Ext. hearing aid	Congenital
IC ≥ 10 ans	17	Female	Ext. hearing aid	CI	Idiopathic
IC ≥ 10 ans	11	Male	CI	Ext. hearing aid	CMV
HS 3–5 ans	5	Male	-	-	-
HS 3–5 ans	5	Male	-	-	-
HS 3–5 ans	4	Male	-	-	-
HS 3–5 ans	4	Female	-	-	-
HS 3–5 ans	3	Male	-	-	-
HS 6–9 ans	7	Male	-	-	-
HS 6–9 ans	6	Female	-	-	-
HS 6–9 ans	9	Female	-	-	-
HS 6–9 ans	9	Male	-	-	-
HS ≥ 10 ans	12	Female	-	-	-
HS ≥ 10 ans	11	Female	-	-	-
HS ≥ 10 ans	16	Female	-	-	-
HS ≥ 10 ans	15	Female	-	-	-
HS ≥ 10 ans	10	Male	-	-	-

BV in blue, CI in orange, and HS in gray, from lightest to darkest 3–5, 6–9, and ≥10 years.

Comparing the results of the GBT gathered with the three above-mentioned populations should reveal the capacity of the test to quantify the balance abilities of children during walking. These results were then compared to their results on the mBOT-2. Additional analyses in which children were clustered in different age sub-groups further assessed the capacity of the test to evaluate the psychomotor development of balance abilities in children.

Note that in Switzerland, CI users are implanted following the guidelines of the workgroup for cochlear implantation of the Swiss ENT society (12). All subjects included in the study were “experienced” CI users, with a period of use of 1–14 years post-implantation. Children wearing external hearing aid(s) had been using the device(s) as soon as possible following the diagnosis of deafness.

### 2.2. Setting

Given the limited existing literature concerning children with BV, a prospective observational exploratory study was conducted to verify the hypotheses detailed earlier. This study was designed in compliance with the guiding criteria for reporting observational studies (STROBE) (10).

The study took place at the Division of ORL and Head-and-Neck Surgery of the Geneva University Hospitals (HUG), from November 2020 to July 2021. Patient recruitment took place in May 2021. The data were collected from May 2021 to June 2021. Data analysis took place from June to July 2021.

### 2.3. Recruitment

The inclusion and exclusion criteria for the study are presented in Table 2. Children diagnosed with BV using the vHIT and/or the caloric test were identified using the clinical databases of the Division of ORL and Head-and-Neck Surgery of the Geneva University Hospitals. Confirmation of BV in these patients according to the diagnostic criteria of the Bárány Society (9) was done at the time of recruitment that took place during the clinical follow-up visits of patients. A total of 13 children were identified in the clinical databases, but two of them were not included since they could not come to the clinic during the study period (between May and July 2021). The pediatric CI users included in this study were selected to be age-matched with BV children. A total of 12 CI were identified as age-matched and meeting the inclusion criteria (see Table 2). One CI subject had to be excluded during the recruitment phase due to a pathological result on the vHIT for one lateral canal. The group of healthy control subjects (HS) was recruited from the outpatient clinic of the Division of ORL and Head-and-Neck Surgery of the Geneva University Hospitals. The HS group was selected to be age-matched to the included BV or CI group and was included in the study after excluding a vestibular disorder by vHIT and a hearing disorder by voice acoumetry.

**Table 2:** Inclusion and exclusion criteria for the study participants.

**General inclusion criteria:** - Ability to walk independently [from 17 months on average (11)].
**Inclusion criteria for children with BV:** - BV diagnosed according to the diagnostic criteria of the Bárány Society (9).
**Inclusion criteria for the CI control group:** - Bilateral or unilateral CI user. - Normal vestibular function documented with the vHIT test (post-implantation; gain ≥ 0.7 for horizontal semi-circular canals). - Age-matched to BV group.
**Inclusion criteria for healthy controls HS):** - Voice acoumetry within the norm (whispered voice understood on both ears). - vHIT ≥ 0,8 gain for horizontal semi-circular canals, both sides. - Age-matched to BV group.
**Exclusion criteria:** - Physical or cognitive disability that prevents understanding or performing the tasks required. - Refusal of the participant or of one of his/her representatives to participate in the study. - Non-compliance with inclusion criteria.

**Figure 1:**
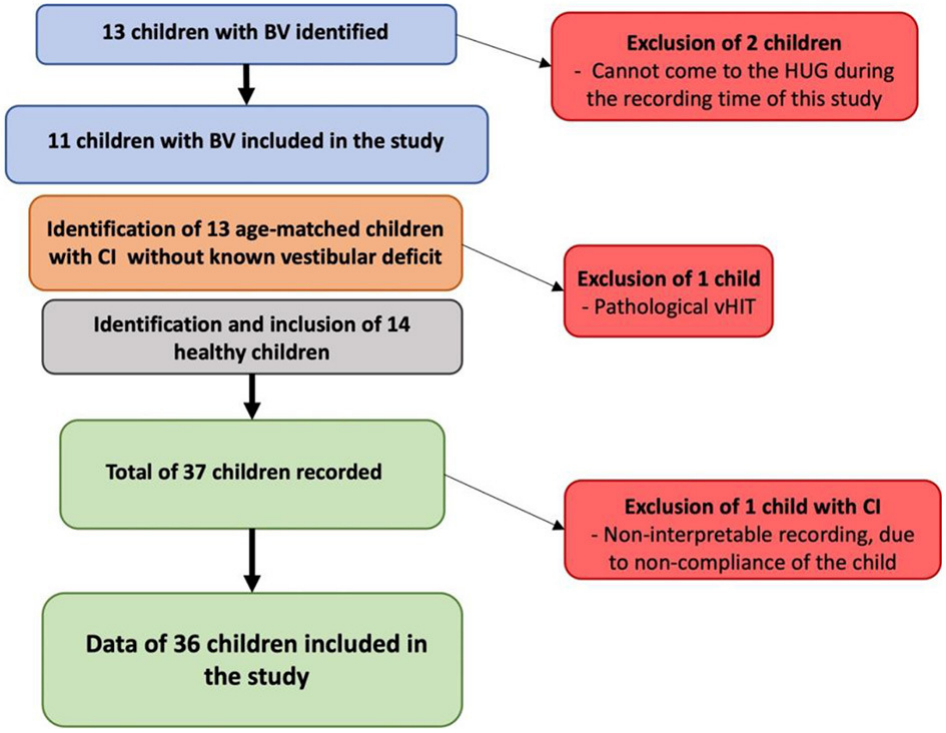
Summary of the recruitment process of the study population and the number of children included.

One subject in the CI group was excluded because of a lack of compliance (the child did not want to perform the GBT or any of the mBOT-2 tasks). Figure 1 below summarizes the recruitment process.

### 2.4. Study procedures

After giving oral information to the parents and collecting oral consent, the children underwent the GBT and the mBOT-2 for ~30 min (5 min for the oral consent, 10 min for the GBT, and 15 min for the mBOT-2). Written information and consent forms were provided and gathered from the parents after study completion for scientific purposes. The procedure, the information forms, and the consent forms have been approved by the Cantonal research ethics commission in Geneva (CCER 2022-00034). None of the participants have been rewarded for their participation, and consent was provided freely.

The GBT was designed to be adapted to children over a broad age range (ideally as soon as they can walk, up to any age), to be cost-effective, and rapidly performable in children. The aim was to isolate the vestibular function from other senses contributing to the balance function (proprioception and vision). To reduce the contribution of proprioception, the tested subject was asked to walk in the middle of a 6 m^*^1 m^*^2 cm foam mat, at a normal walking pace (always with one foot on the ground) in bright light conditions (BL; 45–70 lx), provided by ceiling lights. Then, to reduce the contribution of vision, the same test was performed again in dim light conditions (DL) provided by two punctual lights (KORNSNÖ^®^ LED night light, IKEA, Älmhult, Sweden) on both walls. Red LED biking bracelets (STOKE^®^, Ochsner Sport, Dietikon, Switzerland) were worn by participants on both wrists and both ankles (maximum luminosity 3 lx) to permit visualization of the feet and hands of each participant in DL.

**Figure 2:**
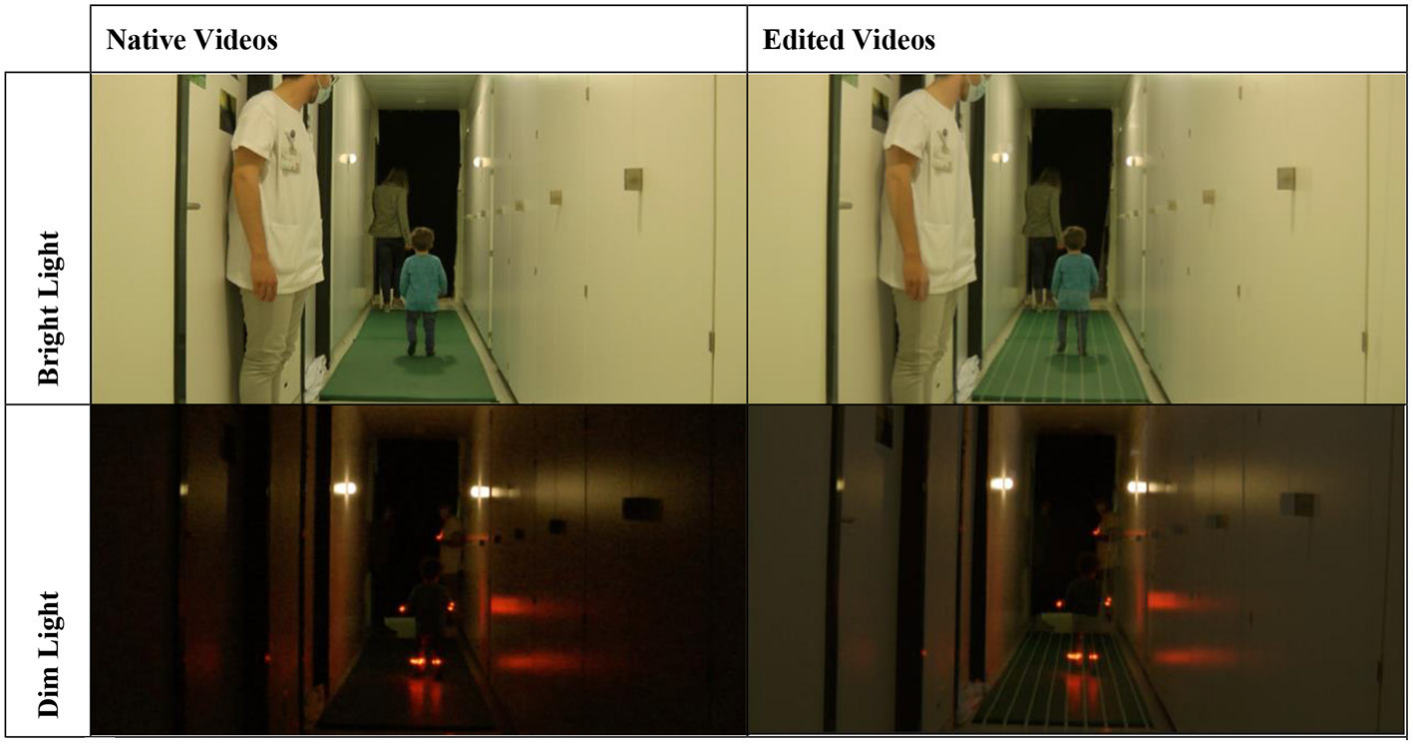
Illustration of the two conditions of the test: bright light—BL **(upper panels)** and dim light—DL **(lower panels)**, before video editing **(left panels)** and after video editing **(right panels)**.

Each test condition was repeated three times and recorded. A camera placed 1 m in front of the mat recorded the entire evaluation. The recorded videos were then superimposed using the iMovie application version 10.2.2.7 (Apple Inc., Cupertino, United States of America) with a reference image showing the same mat with lines spaced 10 cm apart. Once the video and the reference image were superimposed, the transparency of the videos was adjusted to permit a better view of the lines and the subject. The alignment of the two videos could be controlled with the superposition of the two punctual night lights on both walls of the corridor. It was thus possible to measure the deviation to the midline during each walking trial frame by frame, for each subject (Figure 2). The scoring below was used to quantify the deviation in each condition.

0: The subject walks in a straight line, staying in the two central lanes.
1: The subject steps once into the 1st lateral lane.
2: The subject takes several steps in the 1st lateral lane.
3: The subject steps once into the 2nd lateral lane.
4: The subject takes several steps in the 2nd lateral lane.
5: The subject steps once into the 3rd lateral lane.
6: The subject takes several steps in the 3rd lateral lane.
7: The subject steps once into the 4th lateral lane.
8: The subject takes several steps in the 4th lateral lane.
9: The subject uses the walls for support.
The points scored in the right and left lateral lanes were cumulative, for a maximum score of 18 pts if the subject went from one wall to the other.

Note that, for this test, a score of 0 would be representative of perfect balance abilities in a given condition, while higher scores represent worse balance control. Only the run with the best score of the three (i.e., the lowest score) was considered for the BL and DL conditions. The scoring task took ~15 min per subject.

### 2.5. Modified Bruininks-Oseretsky test of Motor proficiency Ed. 2 (mBOT-2)

Participants performed a short, modified version of the balance subtest of the BOT-2 after the GBT. We used a modified version of the BOT-2 as our objective was to perform a short test, bearable for the youngest children (max 30 min overall). The mBOT-2 consists of five out of the six tasks standardly done on the firm ground of the BOT-2, with the repetition on both sides of the one-leg stance [walking on a line feet apart has been excluded of the mBOT-2 as the least sensitive and specific task following Oyewumi (7)]. We did not perform the tasks on the balance beam, as tasks on the firm ground were already unachievable for most of our case subjects. We analyzed the raw results of the following different tasks:

1. Standing on a line with heel to toes (tandem stance), eyes open (9).
2. Standing on a line with heel to toes (tandem stance), eyes closed (9).
3. Standing on one leg, eyes open (9).
4. Standing on one leg, eyes closed (9).
5. Standing on the second leg, eyes open (9).
6. Standing on the second leg, eyes closed (9).
7. Walking forward on a line, heel to toes (tandem walking) (6).

The score for static tasks is calculated by timing the maximum hold time of the required positions. The dynamic test is scored according to the number of steps that the subject can take over the line without deviating. The maximum score for the static tasks is 10 s per task and the maximum score for the dynamic task is six steps, for a total maximal score of 66 points. Therefore, low mBOT-2 scores would be presented in the case of balance problems, and a high mBOT-2 score would be characteristic of good balance skills (i.e., the higher the score, the better the balance skills). All participants had up to two attempts to do the perfect score for each task. The mBOT-2 was filmed using the same parameters as the GBT. The exact timing and the counting of the steps were done frame by frame using the iMovie application, version 10.2.2.7 (Apple Inc., Cupertino, United States of America).

If a task was not completed by the participant, a score of 0 was given for this particular task. The examiner(s) tried to convince each child to perform the task, by miming the asked position or asking several times to do so. All the tests were done in the presence of a parent to ensure a trusting environment.

### 2.6. Statistics

Tests of normality according to Kolmogorov–Smirnov, linearity by point clouds, search for outliers by Mahalanobis distances, and multicollinearity analysis by correlation according to Spearman's Rho were carried out first to verify the suitability of parametric statistical analyses. Since these tests were passed, the scores of the GBT and mBOT-2 were compared among the BV, HS, and CI populations grouped per age ranges of 3–5, 6–9, and ≥10 years using one-way multivariate analysis of variance (MANOVA). If a significant difference was found by the MANOVA, an ANOVA with Bonferroni *post-hoc* tests was used only between significant values, to identify the significant differences.

## 3. Results

**Figure 3:**
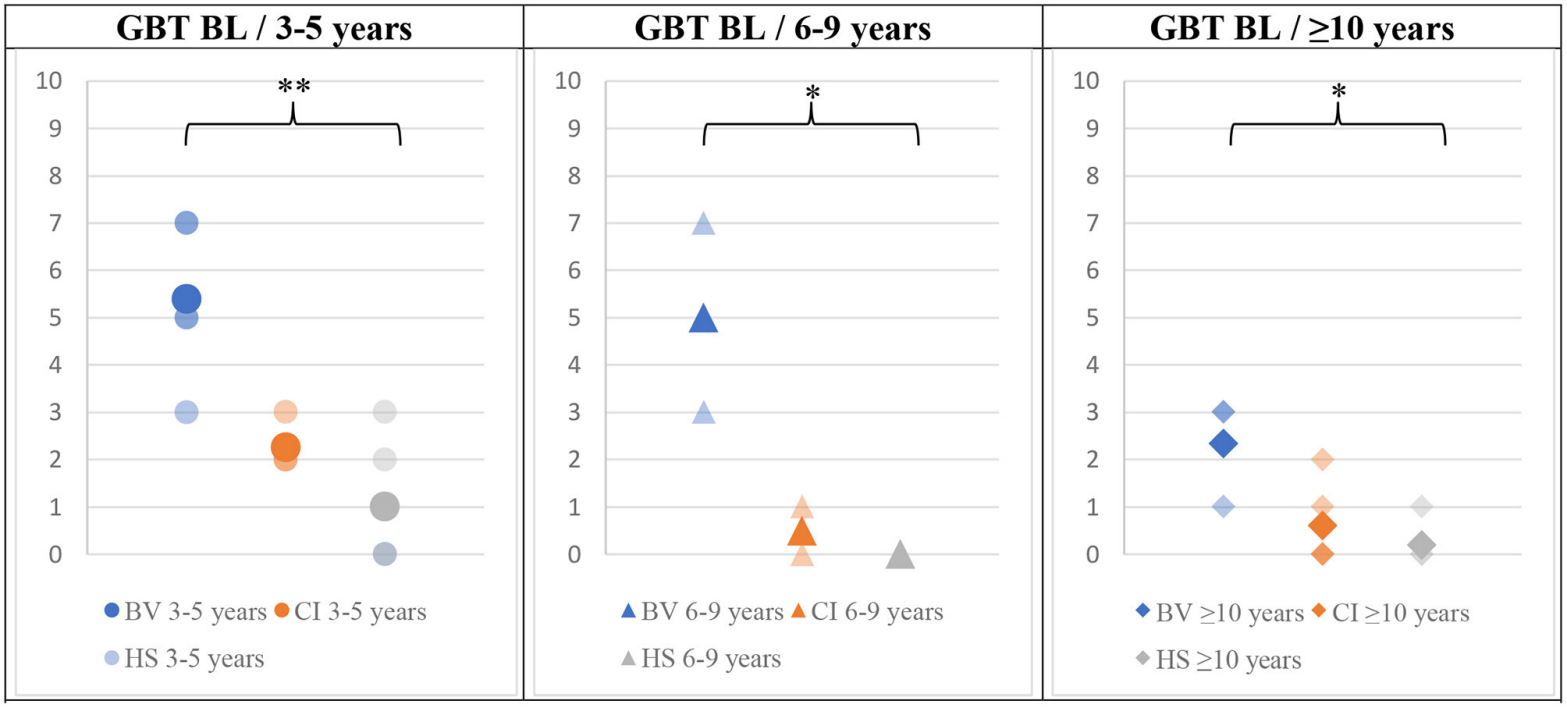
Means (solid symbols) and individual results (semi-transparent symbols) of the GBT in the BL condition, for the three age sub-groups, from left to the right; 3–5/6–9/≥10 years old. *p < 0.017/**p < 0.004 (with Bonferroni correction).

**Figure 4:**
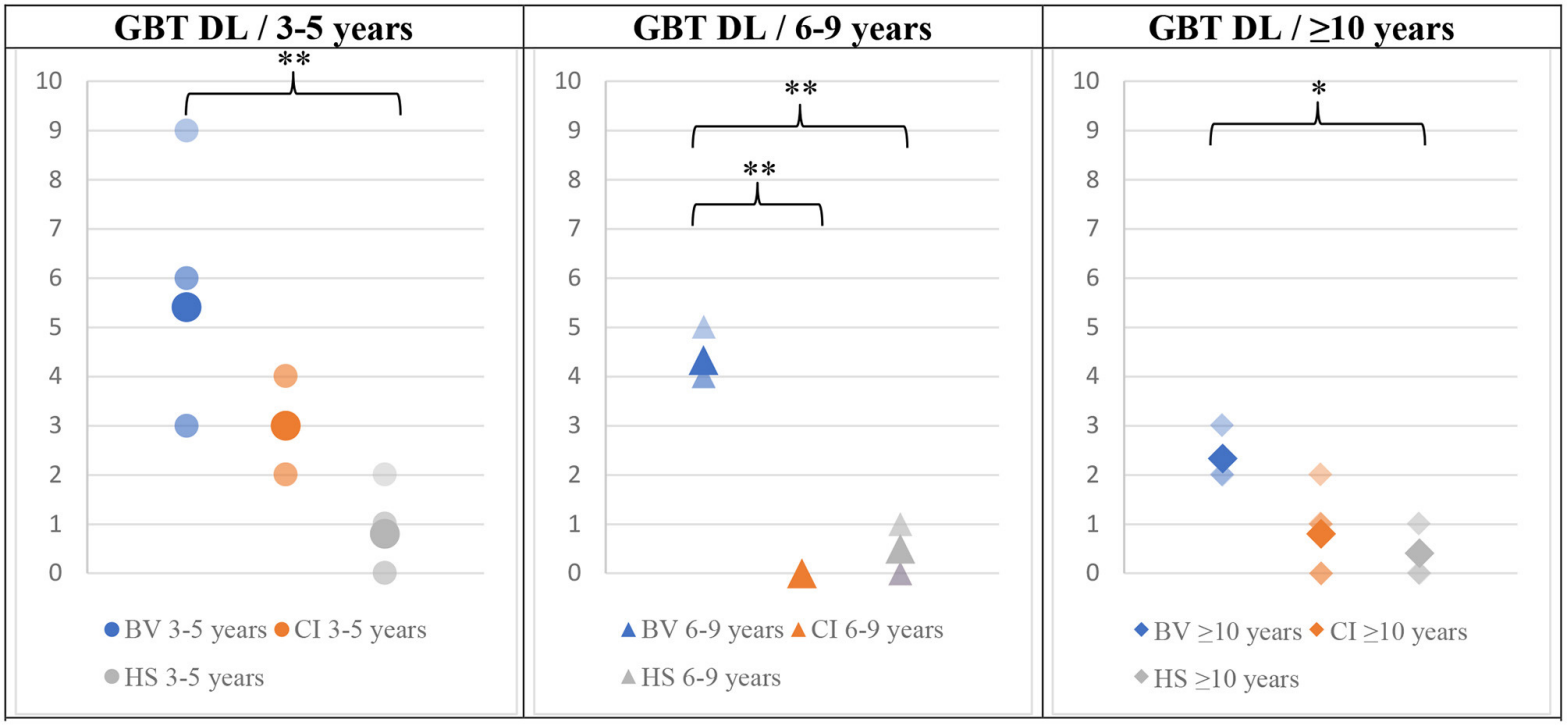
Means (solid symbols) and individual results (semi-transparent symbols) of the GBT in DL for the three age sub-groups, from left to right; 3–5/6–9/≥10 years old. *p < 0.017/**p < 0.004 (with Bonferroni correction).

The mean GBT scores obtained in BL and DL are presented in Figures 3, 4, respectively. BV presented the highest scores (i.e., worse balance abilities) for all age sub-groups (blue symbols). CI (orange symbols) showed higher scores in the youngest 3–5 years sub-group (left panels in the figures), but their scores became similar to HS's results for the two older age sub-groups (6–9 and ≥10 years; middle and right panels in the figures). The HS (gray symbols) showed low scores of 0–1 (close to perfect balance abilities) across all age sub-groups.

**Figure 5:**
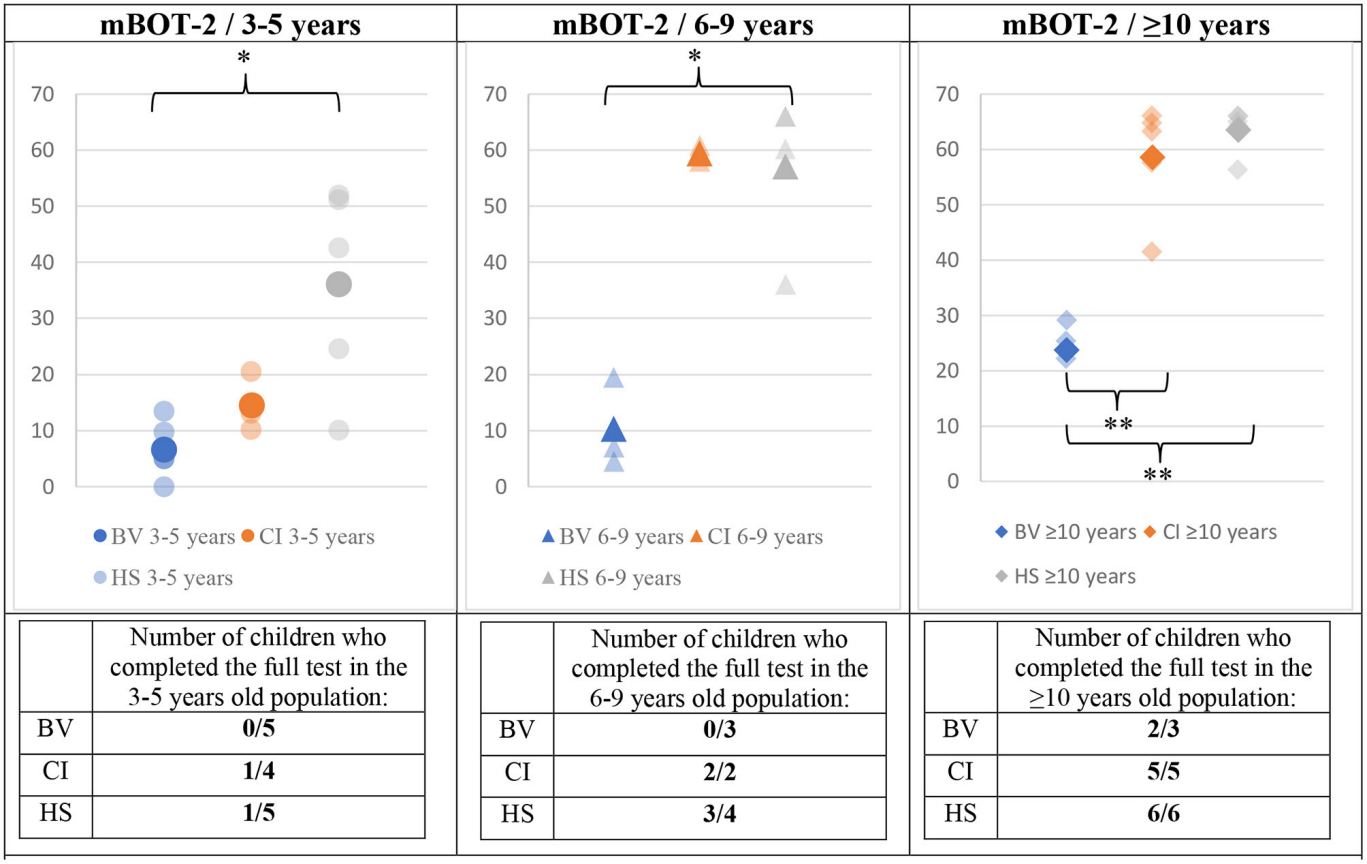
Means (solid symbols) and individual results (semi-transparent symbols) of the BOT2 for the three age sub-groups, from left to right; 3–5/6–9/≥10 years old. The number of children who completed the test (if a task of the test is refused by the subject, a score of 0 is given for that task) is presented in a table below each panel. *p < 0.017/**p < 0.004 (with Bonferroni correction).

The results of the mBOT-2 tests for all age sub-groups are presented in Figure 5. The BV obtained the worst scores across all age sub-groups. CI obtained intermediate scores for the youngest age. For older age sub-groups, CI obtained close-to-perfect scores that even reached the maximum of 66 points for the majority of subjects. The best scores across age sub-groups were obtained by HS, which also saturated the maximum score for the test for the 6–9 and ≥10 years of age sub-groups. The total number of children being able to complete the test in each group is also an interesting outcome (tables below each panel of Figure 5). The reliability of the results and comparisons of the mBOT-2 scores for the youngest age sub-group of 3–5 years is limited since only two out of the total 14 participants (all groups taken together) were able to complete the test. One of the successful participants was in the CI group and the other one was in the HS group. Thus, most children in the CI and HS groups of 6–9 years of age were able to complete the mBOT-2, obtaining scores near the maximum allowed by the test, while all BV were unable to achieve the requested tasks. Finally, for the ≥10 years of age sub-group, all CI and HS were successful in performing the entire mBOT-2, also obtaining close-to-perfect scores. Two out of three BV subjects could also complete the test but scored much poorer than the other two groups.

The Bonferroni-corrected (*p* < 0.017) MANOVA analysis (validated by preliminary statistical analyses, see Methods section) revealed a statistically significant intergroup difference (Pillai's Trace = 1,56 and *p* = 0.03) for the results of the GBT in both BL and DL conditions and for the mBOT-2. When considering the dependent variables separately, the significant between-subjects effects in the younger age sub-group (3–5 years old) were GBT BL (*p* = <0.001), GBT DL (*p* = 0.005), and mBOT-2 (*p* = 0.005). An ANOVA with a Bonferroni *post-hoc* test was then conducted on these three variables that showed significant intergroup differences to identify exactly between which populations the significance exists. The difference was significant only between BV and HS groups, for all tests (GBT BL *p* < 0.001; GBT DL *p* = 0.004; mBOT-2 *p* = 0.006).

In the 6–9 years sub-groups, a statistically significant intergroup difference was also present for the three tests (Pillai's Trace = 1.817 and *p* = 0.0129). When considering the dependent variables separately, the significant between-subjects effects in the 6–9 years sub-group were GBT BL (*p* = 0.004), GBT DL (*p* = <0.001), and mBOT-2 (*p* = 0.003). An ANOVA with a Bonferroni *post-hoc* test was then conducted which revealed a significant difference between BV and HS for all conditions (GBT BL *p* = 0.005; GBT DL *p* < 0.001; mBOT-2 *p* = 0.005) and also for the GBT in DL between BV and CI (*p* < 0.001).

The analysis of the results of the ≥10 years of age sub-groups also revealed a statistically significant intergroup difference (Pillai's Trace = 0.876 and *p* < 0.001). When considering the dependent variables separately, the significant between-subjects effects in this age sub-group were GBT BL (*p* = 0.007); GBT DL (*p* = 0.004), and mBOT-2 (*p* < 0.001). An ANOVA with a Bonferroni *post-hoc* test was then conducted which revealed a significant difference between BV and HS in both BL and DL conditions of the GBT test (BL *p* = 0.015; DL *p* = 0.009). The mBOT-2 results were significant between BV and CI (*p* < 0.001) and between BV and HS (*p* < 0.001).

**Figure 6:**
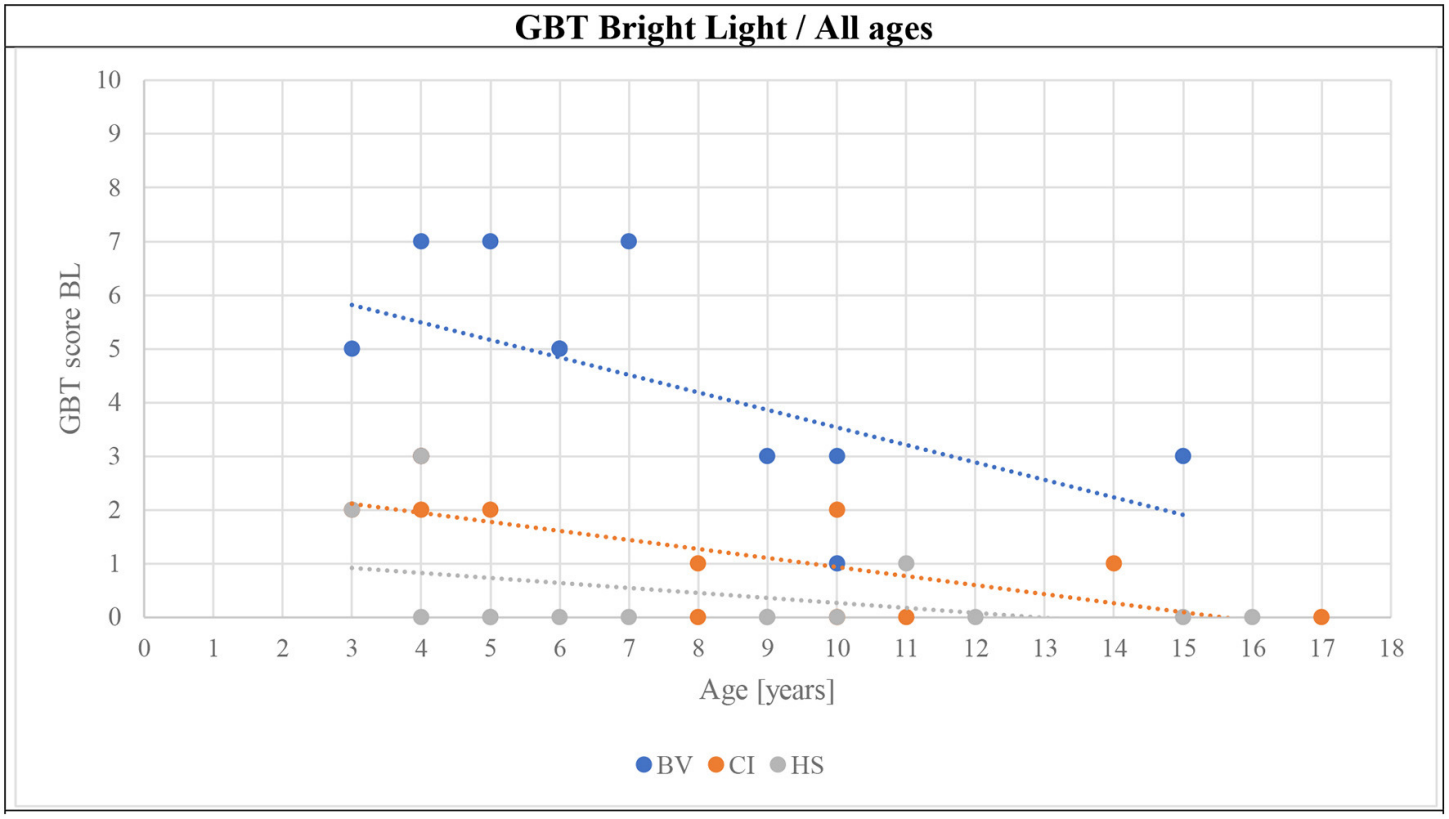
Evolution of the GBT scores of all subjects in BL conditions as a function of age (BV, blue symbols; CI, orange symbols; HS, gray symbols).

**Figure 7:**
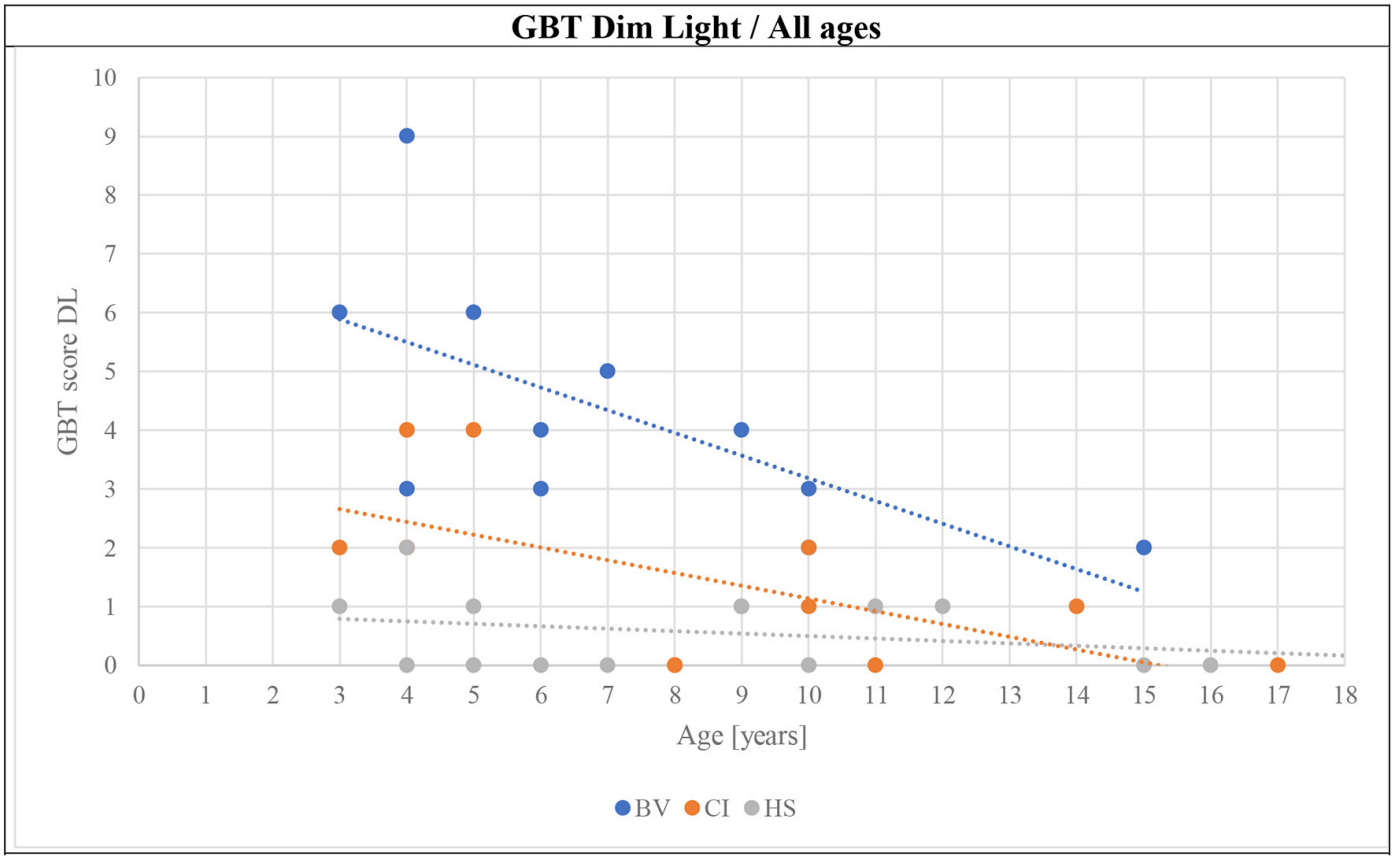
Evolution of the GBT scores of all subjects in DL conditions as a function of age (BV, blue symbols; CI, orange symbols; HS, gray symbols).

**Figure 8:**
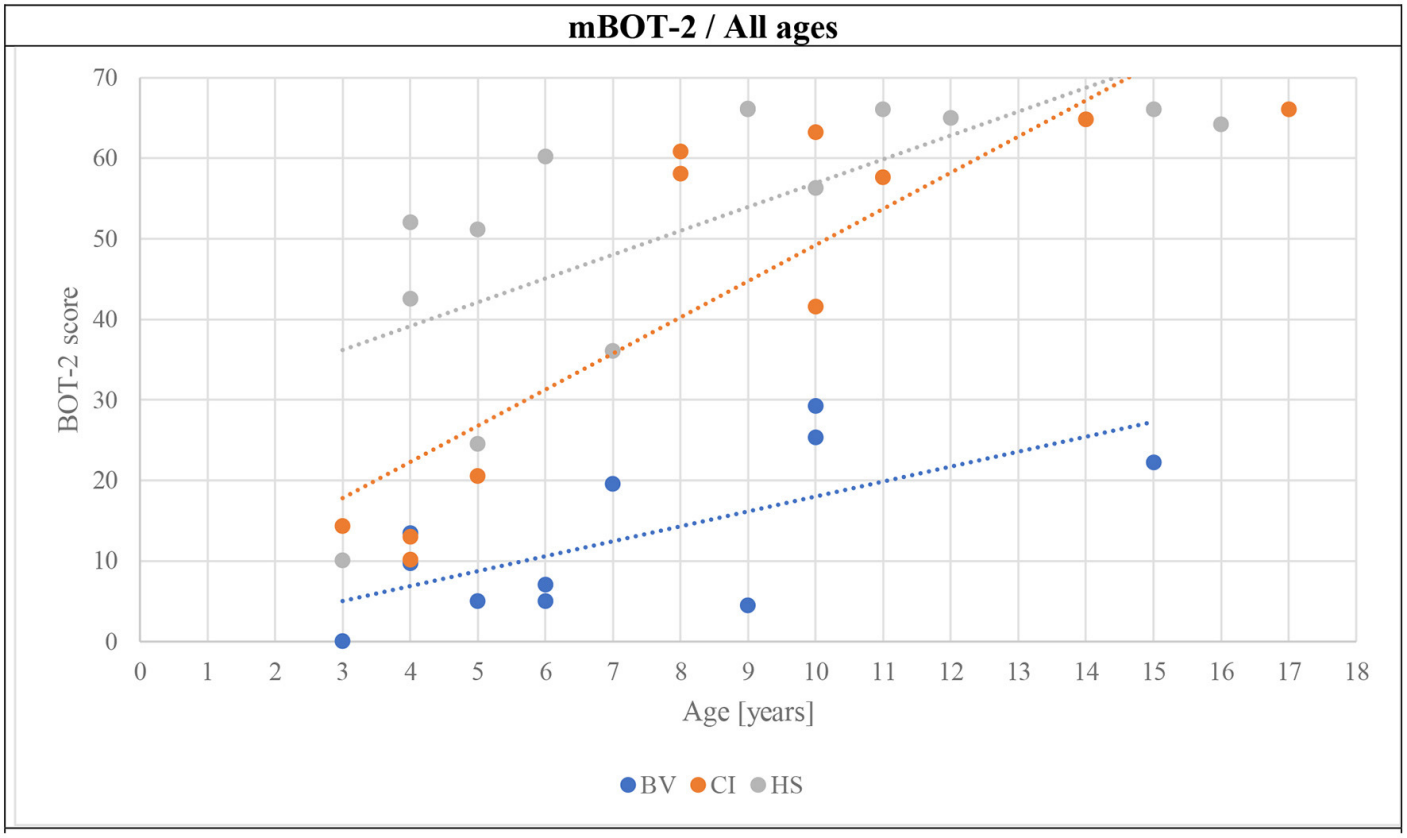
Evolution of the mBOT-2 scores of all subjects as a function of age (BV, blue symbols; CI, orange symbols; HS, gray symbols).

The final hypothesis of this article required the investigation of the evolution of GBT and mBOT-2 scores with age. Figures 6–8 present this comparison. Several observations can be made from these results. First, the scores for the GBT, both in BL and DL, are always higher in BV than in CI and HS groups, at all ages. An improvement in scores with age can be also observed for the three participant groups (BV, CI, and HS), but BV never reaches the performance levels for the other two populations. Interestingly, CI seems to have intermediate scores at young ages, but their scores improve and become comparable to the scores of HS from 7 years old. On the other hand, HS has good scores from the earliest age. Similar observations can be made for mBOT-2 scores: the scores of the BV are poorer than the CI and HS groups, and they remain low even at the oldest ages tested. The youngest CI and HS have low scores that saturate from 5 years. It should be reminded that the BOT-2 is only validated from 4.5 years of age and that only a few children completed the mBOT-2 in the youngest sub-groups.

Finally, a comparison of the GBT in BL and DL and the mBOT-2 results showed a strong correlation between tests [Spearman's Rhô correlation analysis; BL-DL *r*_s_ = 0.891 (*p* < 0.001); BL-mBOT-2 *r*_s_ = −0.787 (*p* = 0.001); DL-mBOT-2 *r*_s_ = −0.732 (*p* < 0.001)].

## 4. Discussion

In the present study, the results suggested that the GBT could be a useful tool for the evaluation of balance capacities in children over a broad age range and for their follow-up. HS obtained high scores on the test from the earliest age. GBT scores for BV were consistently and significantly poorer than for the two control populations included in this study, for all age sub-groups. A small improvement is visible in the BV as a function of age, but they never reached the scores of the control populations. CI presented poorer GBT scores in the 3–5 years age sub-group than their HS counterparts, but this difference seemed to improve with age since the results of older populations were comparable to those of the HS.

The results obtained with the GBT seemed to be in accordance with those obtained with the mBOT-2. BV consistently obtained lower scores. However, our results show that the mBOT-2 is not well-accepted by the youngest children included in this study. Only a few subjects aged 3–5 years completed the test, the interpretation of their results is thus biased. However, these results are concordant with the fact that the BOT-2 is not validated for children <4.5 years of age. The mBOT-2 was performed after the GBT for each child. This could constitute a bias, as children could not achieve it because of tiredness. This bias is limited, as we aimed to perform tests as short as possible.

HS obtained low GBT scores from the 3–5 years age sub-group, presumably reflecting good balance skills starting at an early age. We also observed slight improvements with age. BV presented high scores on this test from the age of 3–5 years, presumably confirming their balance disorder. These children improve their scores with increasing age but maintain poorer scores for ≥10 years old than young 3–5 years old HS. The possibility of observing clear and consistent improvements with age demonstrates the potential of the GBT to monitor the evolution of children's balance abilities with age, which would represent a useful tool that is currently lacking in the clinic for patients over a broad age range. Furthermore, the GBT is shorter to perform than BOT-2 or even the mBOT-2. The mBOT-2 showed saturation of the maximal scores by subjects who are 6–9 years old for HS and CI, which was not the case for the GBT. Moreover, in the specific BV population, the mBOT-2 shows improvement, but the test is poorly accepted and rarely completed. The interpretation of these results in our target population is therefore questionable, with the results of a test that was not completely achieved by the vast majority of BV children.

We also observed that young CI recipients aged 3–5 years had intermediate scores on the GBT, between HS and BV. From the age sub-group of 6–9 years, the scores of the GBT in the CI population reached close-to-perfect scores, which were comparable with the scores of the HS of the same age. This interesting observation tends to the same conclusion as De Kegel et al. (13), who examined the impact of CI on motor development prospectively. It could suggest that deafness or CI might impact the development of balance abilities even in the absence of vestibular deficits. In the small cohort included in this study, it seems that the CI was an effective means to normalize the development of balance abilities with age. The hearing has been indeed shown to play an important role in balance in a healthy population (14). Further studies in a larger cohort are needed to validate this hypothesis and to identify more precisely the influence of CI and/or auditory rehabilitation in the development of balance abilities.

The main limitation of this study is its small sample size. All the BV known in our clinic were included in the study, it was therefore impossible to increase the number of included case patients. A larger scale study would improve the statistical power of the results and would be useful to validate these preliminary findings. In addition, the inclusion of additional pathological populations would also contribute to our understanding of the development of balance abilities in children and help identify potential obstacles and useful tools to improve clinical outcomes at a larger scale.

The age of the children of course influenced the results in terms of normal psychomotor development. It is therefore an effect modifier of our results, which has been diminished by separating the populations into three age sub-groups. The narrower the sub-group of age, the more precise the analysis. A larger scale study would permit to lower this effect. The future use of this test in larger populations could furthermore allow normative results for each age. It could thus define a precise cutoff for each age, reinforcing the screening ability of the GBT.

This project was a preliminary study on the effectiveness of this new clinical tool. The GBT showed some promising results, and the actual setting is perfect in many ways. To allow a more precise definition of the GBT score and an increase in the rapidity of the scoring, an automatic computer-based analysis would be needed. A further study should confirm the transposition of the GBT actual settings with motion capture technology.

## 5. Conclusion

The Geneva Balance Test seems to be a useful tool to contribute to the screening for BV, as they perform significantly lower than the two control groups. The improvement of its scores with age indicates that it could be used in the follow-up of children with BV as well and even to evaluate the effectiveness of therapeutic interventions (e.g., vestibular rehabilitation). The test showed comparable results to the mBOT-2, consisting of most tasks of the balance subunit of the BOT-2 which is validated in the literature. Yet, the GBT seems to be better accepted by young children and allowed to quantify an improvement according to age, which is not easily assessable with the mBOT-2. Finally, an interesting result concerns the improvement in balance abilities of CI users between the two age categories: 3–5 and 6–9 years old. This result could emphasize the influence of CI on balance capacities, even in children without vestibular disorders, but a study with a bigger cohort would be needed to confirm this interesting finding.

## Data availability statement

The original contributions presented in the study are included in the article/Supplementary material, further inquiries can be directed to the corresponding author.

## Ethics statement

The studies involving human participants were reviewed and approved by Commission Cantonale d'Ethique de la Recherche sur l'être Humain de Genève. Written informed consent to participate in this study was provided by the participants' legal guardian/next of kin. Written informed consent was obtained from the individual(s), and minor(s)' legal guardian/next of kin, for the publication of any potentially identifiable images or data included in this article.

## Author contributions

EM, CB, and LB carried out the tests. The inclusion/exclusion criteria were confirmed by CB and EM. CB carried out the vHIT when needed. EM was responsible to confirm the diagnostic of BV following the Bárány criteria, searching for the important information in the patients' database, and wrote the first version of the manuscript. HC, LB, and EM were in charge of the recruitment and communication with the parents and the patients. AP and EM carried out the statistical analysis. CB, MR, J-FC, LB, and EM were in charge of the materials needed for the test. AP and MR were in charge of the engineering needed in this study. All authors contributed to the development of the GBT, planned the experiments, contributed and were proofreaders, and approved the final version of the manuscript.
